# Exosome Therapy: A Promising Avenue for Treating Intervertebral Disc Degeneration

**DOI:** 10.1007/s13770-025-00746-5

**Published:** 2025-08-07

**Authors:** Shreya Bhat, Suresh Kannan, Uday Kumar Kolkundkar, Raviraja Neelavar Seetharam

**Affiliations:** 1 Manipal Centre for Biotherapeutics Research, Manipal Academy of Higher Education, Manipal, Karnataka India; 2 Stempeutics Research Pvt. Ltd., Bangalore, Karnataka 560 048 India

**Keywords:** Exosome therapy, Intervertebral disc degeneration, Stem cells, Nucleus pulposus, Spinal cord injury

## Abstract

**Background:**

The human spine relies on intervertebral discs (IVDs) for support and mobility, functioning as shock absorbers that enable friction-free movement. However, IVDs are susceptible to degeneration (IVDD) due to age, excessive strain, and genetic factors, resulting in bulging or herniation that causes pain, stiffness, and nerve compression.

**Current Treatments:**

Current treatments primarily focus on symptom management through medication, physical therapy, or surgery in severe cases, without addressing tissue repair.

**Emerging Therapies:**

Exosome therapy has recently emerged as a promising regenerative approach for IVDD. Exosomes are small, membrane-bound vesicles released by cells, acting as messengers to transport proteins and RNA that influence recipient cell behavior.

**Potential and Challenges:**

Researchers are investigating exosomes for IVDD because they may promote disc repair and regeneration by delivering molecules that stimulate tissue recovery and carry anti-inflammatory agents to reduce inflammation and modulate pain. Engineering strategies, such as loading exosomes with therapeutic cargo or targeting molecules, can further enhance their efficacy. While exosome therapy for IVDD is still in early research stages, ongoing studies are promising, though challenges remain in optimizing isolation methods and ensuring clinical safety.

**Conclusion:**

Exosome-based therapies could offer a safe, effective, and minimally invasive solution for individuals affected by IVDD.

## Introduction

An intervertebral disc (IVD) is a fibrocartilage cushion between vertebrae, providing flexibility and shock absorption. Humans have 23 discs: six cervical, twelve thoracic, and five lumbar [1]. Each disc consists of three regions: the nucleus pulposus (NP), annulus fibrosus (AF), and cartilaginous endplate (CEP) [2]. The NP, a gel-like structure at the center, is crucial for spine strength and flexibility, and is composed mainly of chondrocyte-like cells, type II collagen, proteoglycans, and water. It contains a low density of specialized cells (chondrocyte-like and notochordal cells) that maintain the extracellular matrix (ECM). A ring-shaped structure called the AF, a highly fibrous structure encircles the NP. The CEP connects the IVD to the vertebral body, consisting of a hyaline cartilage zone near the disc and a calcified zone attached to the bone. This structure provides a strong yet flexible attachment, anchoring the disc and facilitating efficient load transfer during movement [3].

Any disturbances in these normal functions of the disc cause intervertebral disc degeneration (IVDD). It leads to a cascade of structural and biochemical changes significantly impacting spinal health. The degeneration is characterized by loss of hydration, cell death, and altered mechanical properties, which can result in pain and disability in the disc. The following sections outline the key consequences of IVDD [4]. While current treatments like analgesics, physical therapy, and invasive surgeries provide symptomatic relief, they fail to address underlying biological mechanisms and often entail significant risks of complications or recurrence. This therapeutic gap underscores the need for regenerative strategies targeting IVDD's molecular underpinnings [5]. Extracellular vesicles (EVs) are membrane-bound particles released by cells into the extracellular environment and include several subclasses, such as exosomes, microvesicles, and apoptotic bodies. Exosomes are a specific subset of EVs, typically 30–150 nm in diameter, originating from the endosomal pathway. In this manuscript, the term “EVs” is used when referring to the broader class of vesicles, while “exosomes” specifically denotes this well-characterized subclass. Exosomes, particularly those derived from mesenchymal stem cells (MSCs), have emerged as a promising avenue for IVDD treatment. These nanoscale vesicles facilitate intercellular communication and possess regenerative properties that can counteract the detrimental effects of degeneration. Research indicates that stem cell-derived exosomes can inhibit apoptosis, modulate inflammatory responses, and promote the regeneration of nucleus pulposus cells (NPCs). By leveraging their ability to deliver bioactive molecules and maintain homeostasis within the disc environment, exosomes represent a novel approach to enhance disc repair and regeneration while addressing limitations in current treatment modalities [6].

## Etiology of intervertebral disorders

IVDD results from a complex interplay of various factors disrupting the balance between extracellular matrix synthesis and degradation [7]. A study from 2018 found that the prevalence of lower back pain caused by disc disease was 3.36% worldwide [8]. IVDD sits at the core of this narrative. It can be described as a loss of disc volume, primarily affecting the NP and AF. This loss of volume disrupts the functional integrity of the spine, its ability to distribute pressure, and its role as a shock absorber [9]. The degeneration process can be attributed to a combination of different types of stress, genetic predisposition, etc.

### Mechanical overload and structural compromise

Chronic mechanical overload from heavy lifting or poor posture causes tears in the AF and dehydration of the NP, weakening IVDs and increasing pain vulnerability with age [10]. A study by Gawri et al*.* showed that human IVD cells subjected to chronic mechanical stress exhibited increased expression of genes *TLR4, TLR2, TNFα*, and *NGF*, encoding inflammatory mediators associated with back pain [11]. Immobilization can harm IVDs by causing hypomobility, leading to matrix remodelling and further degeneration. The ECM, rich in proteoglycans that retain water for hydration and flexibility, weakens with age and mechanical stress, exacerbating disc issues. This loss disrupts the matrix's structure and signalling pathways, leading to increased cross-linking and stiffening of the disc tissue. Consequently, the disc becomes less adaptable to daily movements and more susceptible to damage at the CEP and AF, ultimately contributing to IVDD [12].

### The role of genetics

Genetic predisposition significantly influences IVDD, with studies identifying specific genes and markers linked to increased risk through heritability. These genes encode proteins crucial for maintaining a healthy disc matrix, such as collagens (*COL11A1, COL1A1*) [13]. However, variations of single nucleotide polymorphism (SNPs) within these genes can disrupt these proteins' function, while other SNPs identified code for enzyme matrix metalloproteinases (MMPs) that break down the matrix. This highlights how genetic variations can compromise the disc's structural integrity and contribute to IVDD pathogenesis. Beyond genetic variations, epigenetic modifications such as DNA methylation and histone changes, influence gene expression in disc cells, adding complexity to IVDD development. Animal models help explore genetic factors by manipulating specific genes to identify potential therapeutic targets for IVDD [14].

### Inflammation

In healthy IVDs, resident disc cells and infiltrating immune cells, such as monocytes and dendritic cells, maintain homeostasis by producing inflammatory mediators like TNFA and IL-1β [15]. However, excessive production of these mediators in degenerated discs leads to chronic inflammation, promoting nerve ingrowth and pain. Additionally, infections and tissue injuries can trigger adaptive inflammatory responses, with chronic inflammation resulting from a failed acute response, where the body's initial attempt to heal from an injury or infection becomes persistent [16].

This highlights the importance of resolving inflammation effectively, as it can contribute to the development of painful conditions like those arising from degenerated discs [17]. There are three common types of disc disorders. The first one is herniated discs that feature a bulging NP that compresses nerves, causing leg pain due to inflammation and pressure from the protrusion. Secondly, degenerative disc disease (DDD) that gradually weakens discs due to reduced nutrients, mechanical stress, and cell death, causing sporadic neck or back pain where non-surgical treatments can often reverse the damage [18].

## Current treatment options for IVDD

Current therapies for IVD diseases involve a combination of non-surgical methods, surgical methods, and biological and cellular therapies. These methods include the following:

### Medications

Analgesics, muscle relaxants, and non-steroidal anti-inflammatory drugs (NSAIDs) which reduce pain and inflammation, are typically recommended for short-term use. Exercise, heat/cold therapy, acupuncture, massage, hydrotherapy, and shock waves are advised for chronic pain. For faster recovery, steroids are usually considered alongside other treatments. Physical therapy improves disc flexibility and strengthens muscles, reducing discomfort and swelling. Injection therapy, often the first choice due to its minimally invasive nature, delivers targeted pain relief and involves delivering medication directly into a specific location within the body using a needle and syringe. corticosteroidal injections are generally used and injected into the epidural space for it to reach the inflamed tissue. This approach eases discomfort but may not fully address the root cause [19].

### Surgical methods

When conservative therapy fails, surgery offers a definitive solution, but traditional approaches can be disruptive. Minimally invasive endoscopic techniques are emerging as a promising alternative [20]. This procedure entails excising the damaged tissue, such as a disc or hypertrophic ligament pressing on a nerve, to give the patient a long-term solution [21]. Corrective operations have been shown to lower the incidence of disc degeneration in a comparison between patients with and without surgery over a 10-year post-operative follow-up study. Despite reducing disc degeneration, surgery itself can weaken the disc due to manipulation and removal of herniated material [22].

### Cell therapy

To promote healing and lessen discomfort, this method entails infusing cells such as MSCs, pluripotent stem cell-derived cells, and IVD-derived stem cells intradiscally into the damaged disc. MSCs, a promising cell therapy for IVDD, may promote tissue repair and pain relief, although their exact mechanism remains unclear [23, 24]. A pilot study utilizes autologous bone marrow MSCs (BMSCs) as a treatment for ten patients with lumbar disc degeneration. This year-long study injected MSCs into patients' backs, leading to reduced pain and disability. This approach shows promise compared to other surgeries [25]. Despite promise as an alternative to surgery, cell therapy faces hurdles: immune rejection risk and the harsh disc environment that limits cell survival and function. Hence, further research is needed to improve cell therapy for IVDD, enhancing the disc environment, cell differentiation methods, and carrier materials [15].

### Biomaterials

This involves implanting a biocompatible material into the affected disc to promote healing and reduce pain. Hydrogels are gaining interest for IVDD due to their biocompatibility, injectability, and ability to support disc cell growth while allowing customization and incorporation of bioactive substances for tissue repair. Clinical trials in the past three decades have been done on scaffold-based injectable therapies to reverse the symptoms of IVDD [26]. Various biomaterials, including natural polymers like collagen and synthetic polymers like polylactic-co-glycolic acid (PLGA), are used for scaffold preparation to aid AF regeneration. Methods include 3D bioprinting, electrospinning, and decellularized scaffolds in tissue engineering [27]. Research focused on soft biomaterials and cell therapies for IVDD aims to achieve biological and biomechanical repair, which targets tissue repair and pain management. These techniques are still under investigation [26].

### Gene therapy

Gene therapy is a promising early-stage IVDD treatment, modifying disc cell genes to increase the production of molecules essential for disc repair, directly targeting the root cause. For instance, *SOX9* gene therapy is being explored as a prospective treatment for IVDD. This approach targets the *SOX9* gene, which is crucial in regulating the balance between anabolic and catabolic processes within the IVD, potentially offering a novel strategy for restoring disc health and function [28]. Another promising IVDD treatment involves injectable DNA hydrogels loaded with the miR-5590-SNA gene, targeting the disc's microenvironment to inhibit cell death and regulate essential component production, potentially slowing IVDD progression [29]. Tools like CRISPR-Cas systems (for genome editing) which utilize RNA interference (RNAi) (for gene silencing) are also being used as a gene therapy to silence specific genes involved in IVDD progression [30].

## Exosome origins, therapeutic applications, and mechanisms of action

**Fig. 1:**
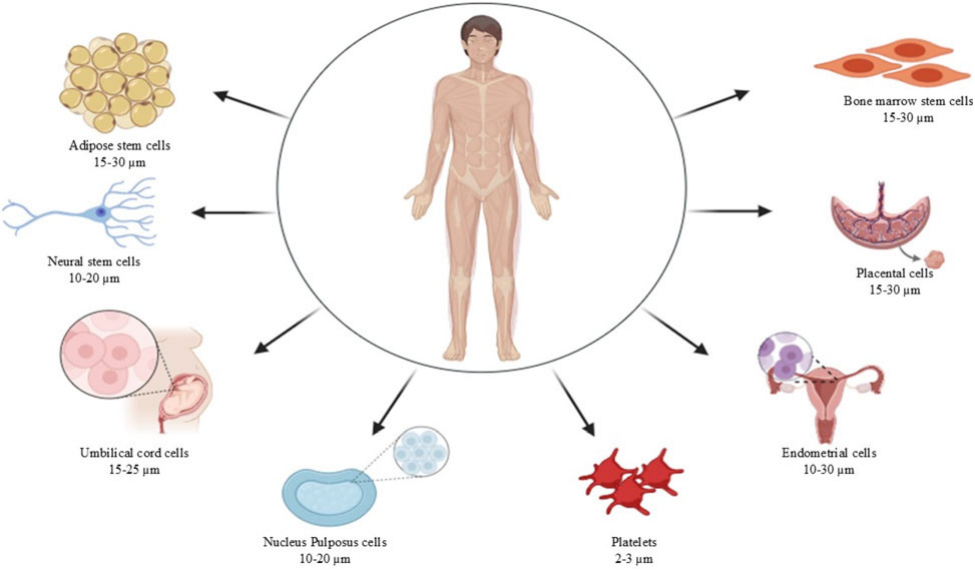
Sources of exosomes and their associated cell sizes in relation to exosome dimensions. Exosomes, nanosized extracellular vesicles ranging from 30 to 150 nm, are secreted by various cell types and play a crucial role in intercellular communication. This figure illustrates the diverse biological sources of exosomes, including adipose stem cells (15–30 µm), neural stem cells (10–20 µm), umbilical cord cells (15–25 µm), nucleus pulposus cells (10–20 µm), platelets (2–3 µm), bone marrow stem cells (15–30 µm), placental cells (15–30 µm), and endometrial cells (10–30 µm). The inclusion of cell sizes provides context for understanding the relative scale difference between the originating cells and the exosomes they produce, emphasizing the nanoscale size of exosomes compared to their parent cells. This comparison underscores the precision and efficiency of exosome-mediated signaling despite their small size relative to the source cells. (made from Biorender)

Exosomes, nanosized vesicles derived from various cells like mesenchymal and neural stem cells, and other sources like mentioned in Fig. 1 hold immense therapeutic potential. Their cargo—proteins, RNA, and lipids—enables functions such as tissue regeneration and neuroprotection [31]. In comparison to larger EVs such as microvesicles (100–1000 nm) and apoptotic bodies (> 1000 nm), exosomes have a unique advantage in their inherent ability to traverse biological barriers such as the blood–brain barrier because of their size and biocompatibility. Such selective accessibility is really a game-changer for drug delivery. Exosomes have the potential to deliver therapeutic cargo more selectively to target tissues, reducing off-target effects compared to some traditional medications. This reduces unwanted side effects and maximizes the effectiveness of the treatment [32].

**Fig. 2:**
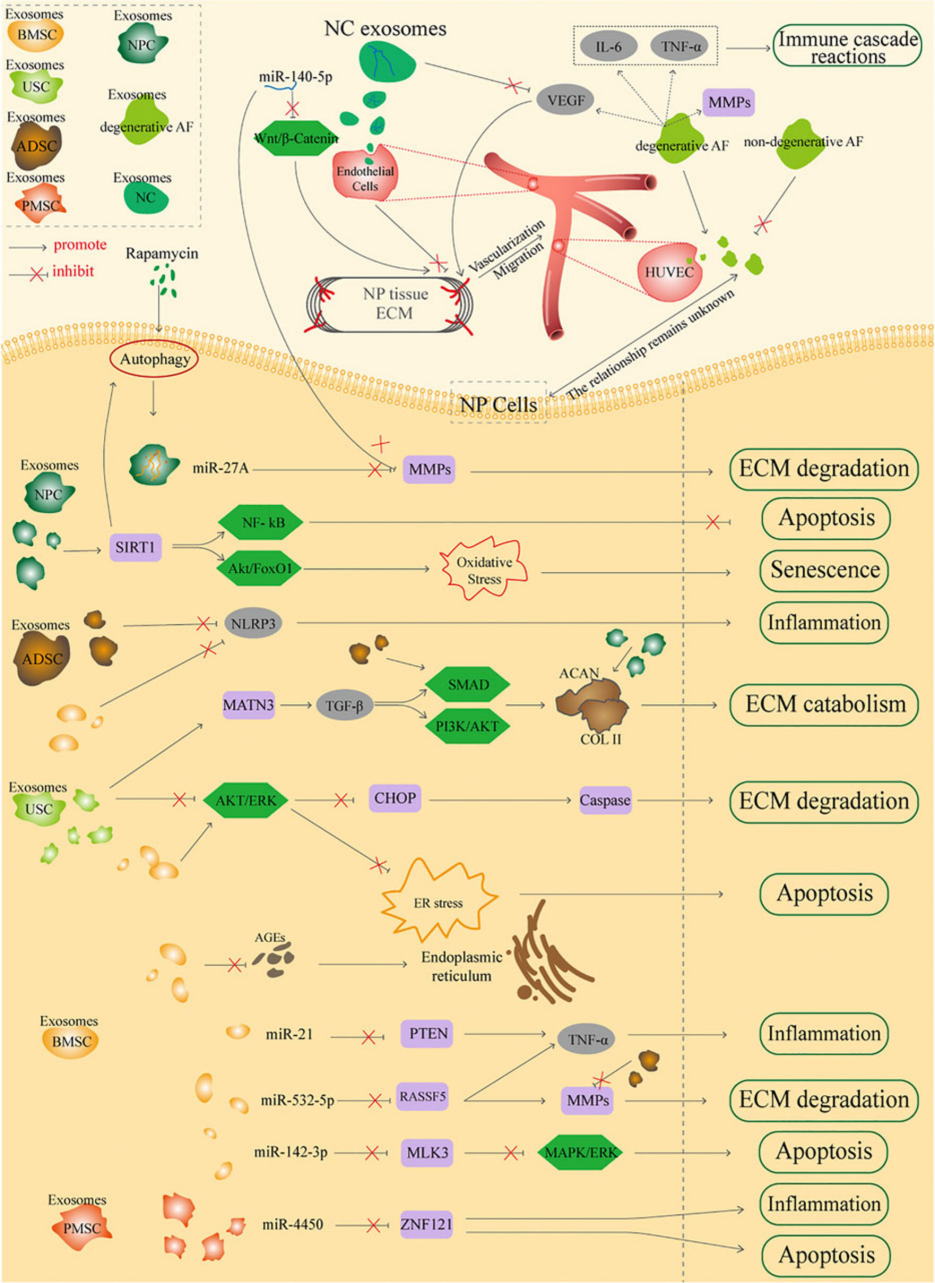
This schematic illustrates how exosomes derived from various cell types-including nucleus pulposus cells, notochordal cells, and mesenchymal stem cells from bone marrow, adipose tissue, umbilical cord, and placenta-modulate key processes in IVDD. Through delivery of miRNAs and proteins, these exosomes regulate extracellular matrix homeostasis, inhibit apoptosis, reduce inflammation, suppress ER stress, and promote regeneration of disc cells, collectively contributing to the attenuation of IVDD progression and offering promising therapeutic potential. Adapted from "Exosomes Immunity Strategy: A Novel Approach for Ameliorating Intervertebral Disc Degeneration" by Li et al., 2022, Frontiers in Cell and Developmental Biology, CC BY 4.0. https://doi.org/10.3389/fcell.2021.822149

Moreover, the source-dependent functionality of exosomes opens doors to personalized medicine, as exosomes exert their therapeutic effects through specific molecular pathways tailored by their cell of origin. For example, miRNAs like miR-146a and miR-181 carried by MSC exosomes suppress pro-inflammatory cytokines (e.g., IL-1β, TNFA) while enhancing M2 macrophage polarization [33]. Neural Stem Cell (NSC)-derived exosomes activate autophagy through miR-374-5p-mediated *STK4* suppression and inhibit neuronal apoptosis via *YY1* pathway modulation by miR-219a-2-3p. These mechanisms highlight how exosomal cargo directly influences cellular repair processes [34]. Such mechanisms highlight the direct regulation of the cellular repair process by exosomal content. As shown in Fig. 2 below [35], exosomes can be obtained from a variety of biological sources, highlighting their widespread presence.

**Fig. 3:**
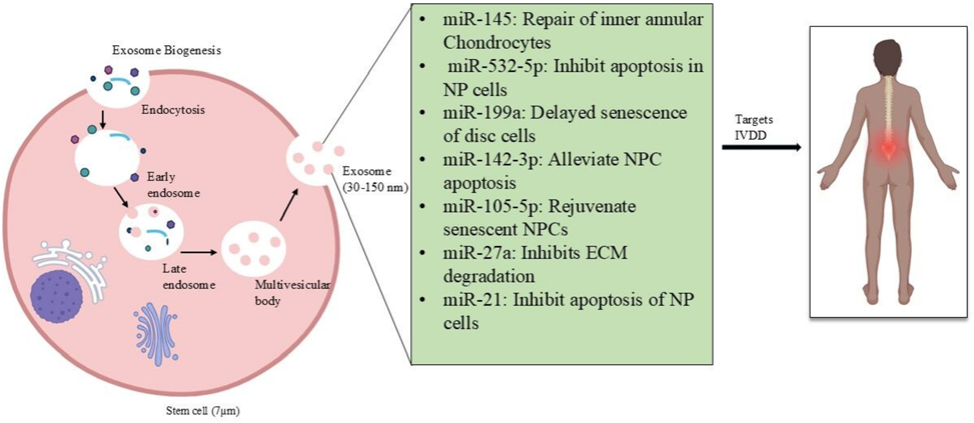
The figure depicts exosome biogenesis, highlighting the formation and encapsulation of miRNAs. The process begins with endocytosis, followed by the formation of early and late endosomes, which mature into multivesicular bodies. These bodies then release exosomes containing specific miRNAs, such as miR-145 and miR-532-5p, which target IVDD. (made from Biorender)

Exosomes are produced inside cellular compartments called multivesicular bodies (MVBs). These MVBs bulge inwards, creating tiny pockets filled with cellular cargo. Eventually, the MVB fuses with the cell's outer membrane, releasing the exosomes into the surrounding environment [36]. Exosomes enter the extracellular space to either be internalized by recipient cells or interact with cell surface receptors [37]. Endocytosis is the primary mode of uptake of exosomes, followed by endosomal release to transfer bioactive cargoes into the cytoplasm. During this process, proteins, lipids, and nucleic acids within exosomes are able to trigger changes in gene expression, modulate signaling pathways like Mitogen-Activated Protein Kinase (MAPK) or oxidative stress response, and mediate anti-apoptotic effects within target cells. Surface proteins on exosomes also interact with target cell-specific receptors for selective therapy molecule delivery for intercellular communication and signal transduction [38]. Various types of stem cells, dendritic cells, cancer cells, macrophages, epithelial cells, and neural cells are known to secrete exosomes [39]. Their production extends beyond the cellular level [40] as they’re found in bodily fluids like urine, serum, blood, synovial fluids, and amniotic fluids [41]. Exosomes are exciting for regenerative therapies due to their ability to carry diverse biomolecules and their small size, which enables them to cross the blood–brain barrier, offering promising advantages for treating various diseases [42]. This targeted delivery system is versatile, as it holds immense potential for curing affected tissues with minimal side effects [43]. Figure 3 below shows therapeutic miRNA present in exosomes that have been proven to alleviate IVDD [44–46].

Research shows that EVs from expanded human umbilical cord blood CD133 + cells and bone marrow MSCs can induce angiogenesis and migration, highlighting their therapeutic potential [47]. The cargo composition of exosomes varies significantly depending on their cellular origin. For example, MSC-derived exosomes contain immunomodulatory miRNAs like miR-146a and miR-181, which suppress pro-inflammatory cytokines (IL-1β, TNFA) and promote anti-inflammatory M2 macrophage polarization in retinal degeneration models [48]. Moreover, NSC-derived exosomes are enriched with miR-219a-2-3p, which inhibits neuronal apoptosis via the *YY1* pathway in spinal cord injury, and miR-374-5p, which activates autophagy through *STK4* suppression [34].

The next generation of exosome therapy shows promise for targeted treatments, as modified exosomes can be engineered to release higher concentrations of therapeutic agents. For instance, engineered exosomes fused with drug-loaded thermosensitive liposomes demonstrated significant drug release under laser irradiation, enhancing cancer treatment efficacy [49]. There has also been an increase in exosome-based early clinical trials for conditions like neurodegenerative diseases, cancers, diabetes, etc. Most of these exosomes have been sourced from MSCs, dendritic cells, plasma, etc. [50]. Henceforth, they have a great possibility of attaining success in the trials and replacing conventional medicines shortly. Table 1 below shows exosomes from different sources, their mode of action, and therapeutic benefits in the context of IVDD in animal models.

**Table 1:** List of animal studies on Exosomes derived from different cellular sources, their pathway, and their therapeutic effect

Cellular source of exosomes	Mode of action	Therapeutic effect	References
Umbilical cord derived MSCs	Reduction in pro-inflammatory cytokines, TNFA, IL-1β, and IL-6 Downregulation in the expression of MMP13 and ADAMTS5 in chondrocytes	Promotion of Regeneration of Articular Cartilage in Rat Models of Osteoarthritis (OA)	[51]
Adipose tissue derived MSCs	Inhibition of inflammatory pathways like the NLRP3 inflammasome, reducing cell death and promoting neuronal survival	Improved spinal cord injury (SCI) repair in animal models by inhibiting NLRP3 inflammasome activation	[52]
MSCs	Exosomes loaded with PTEN siRNA promote axonal regeneration and functional recovery after complete spinal cord injury by inhibiting PTEN, which activates the PI3K/Akt/mTOR pathway and reduces inflammation in the injured area	In mice, delivery of MSC-derived exosomes loaded with PTEN siRNA significantly enhanced motor function following complete SCI compared to control groups	[53]
Pericytes	Pericyte-derived exosomes maintain blood-spinal cord barrier integrity, reduce inflammation by modulating apoptotic factors, and regulate hypoxia-inducible factors, promoting recovery after spinal cord injury	Improved microcirculation and protection of the blood-spinal cord barrier following SCI in mice leading to improved motor control	[54]
Human placenta-derived MSCs	Promotion of angiogenesis and neuroprotection by delivering growth factors that enhance endothelial cell function, improve blood flow, reduce inflammation, and facilitate recovery after spinal cord injury	Significant improvements in neurological function following SCI in mice models. The treatment led to enhanced motor recovery and reduced neurological deficits	[55]
Human placenta-derived MSCs	Human placenta MSC-derived exosomes can activate endogenous neurogenesis, which involves the proliferation and differentiation of neural progenitor cells to replace damaged neurons and promote functional recovery after SCI	Significant improvement in functional recovery and enhanced activation of endogenous neurogenesis following SCI in animal models	[56]
Adipose tissue-derived MSCs	Exosomes derived from these cells inhibit matrix metalloproteinases and promote collagen II synthesis, improving ECM balance in degenerating discs	Improved disc height and reduced degeneration in a rat model of IVDD	[57]
MSCs	Exosomes deliver miRNAs that modulate endoplasmic reticulum stress pathways, reducing apoptosis and promoting the survival of NPCs, thereby ameliorating IVD degeneration	Protection of NPCs from apoptosis, promoting survival, and ameliorating IVDD progression in a rat model	[58]
CEP derived stem cells	CESC-derived exosomes activate the Akt signaling pathway in NPCs, enhancing autophagy and reducing apoptosis, which protects against degeneration and promotes cellular health in IVDs	Inhibition of IVDD in rats by protecting NPCs from apoptosis and promoting their survival	[59]
BMSCs	Promote autophagy in NPCs, inhibit inflammatory factors like IL-1β and TNFA, and suppress apoptosis through the Akt-mTOR pathway	Alleviation of IVD degeneration in rat models by enhancing NPCs survival and reducing inflammation	[60]

## Exosomes in clinical application for IVDD

Exosomes, tiny cellular messengers, have emerged as a promising therapeutic avenue for IVDD in preclinical studies. However, translating this promise into real-world patient care remains a hurdle. While research suggests their potential, clinical applications of exosomes for IVDD are still in their early stages.

Highlighting the limited clinical translation, there has only been one documented clinical trial using exosomes for IVDD treatment so far. Fortunately, the field is not stagnant. Currently, there is a controlled, randomized, double-blind clinical trial that has been completed in India for which the results are yet to be published. This trial investigates the efficacy of exosome therapy for IVDD. This phase 1 clinical trial (NCT04849429) focuses on injecting a patient's own platelet-rich plasma [PRP], enriched with exosomes, directly into the NP of the degenerated disc for chronic low back pain. The results of this trial are crucial in determining the potential of exosome therapy as a viable treatment option for IVDD patients [61]. Another study says, BMSC-derived EVs loaded with circular RNA (circ_0072464) inhibited NPC ferroptosis, promoted matrix synthesis and proliferation of NPCs, and alleviated IVDD in mice by regulating the circ_0072464/miR-431/NRF2 pathway. This suggests that BMSC-EVs carrying mi could be a potential therapeutic target against IVDD [62].

In a promising development for IVDD treatment, researchers investigated the therapeutic potential of exosomes derived from BMSCs. Their study suggests that BMSC-exosomes may offer a novel strategy to halt the progression of IVDD. The researchers found that BMSC-exosomes are enriched with a specific circular RNA molecule, circ_0050205. This circular RNA appears to promote the survival of vital cells within the disc NPCs and suppress the degradation of the ECM, a key structural component. By supporting NPC health and maintaining disc integrity, BMSC-exosomes loaded with circ_0050205 have the potential to impede IVDD progression, offering a promising therapeutic avenue for future research [63].

## Role of exosomes in disc homeostasis

**Fig. 4:**
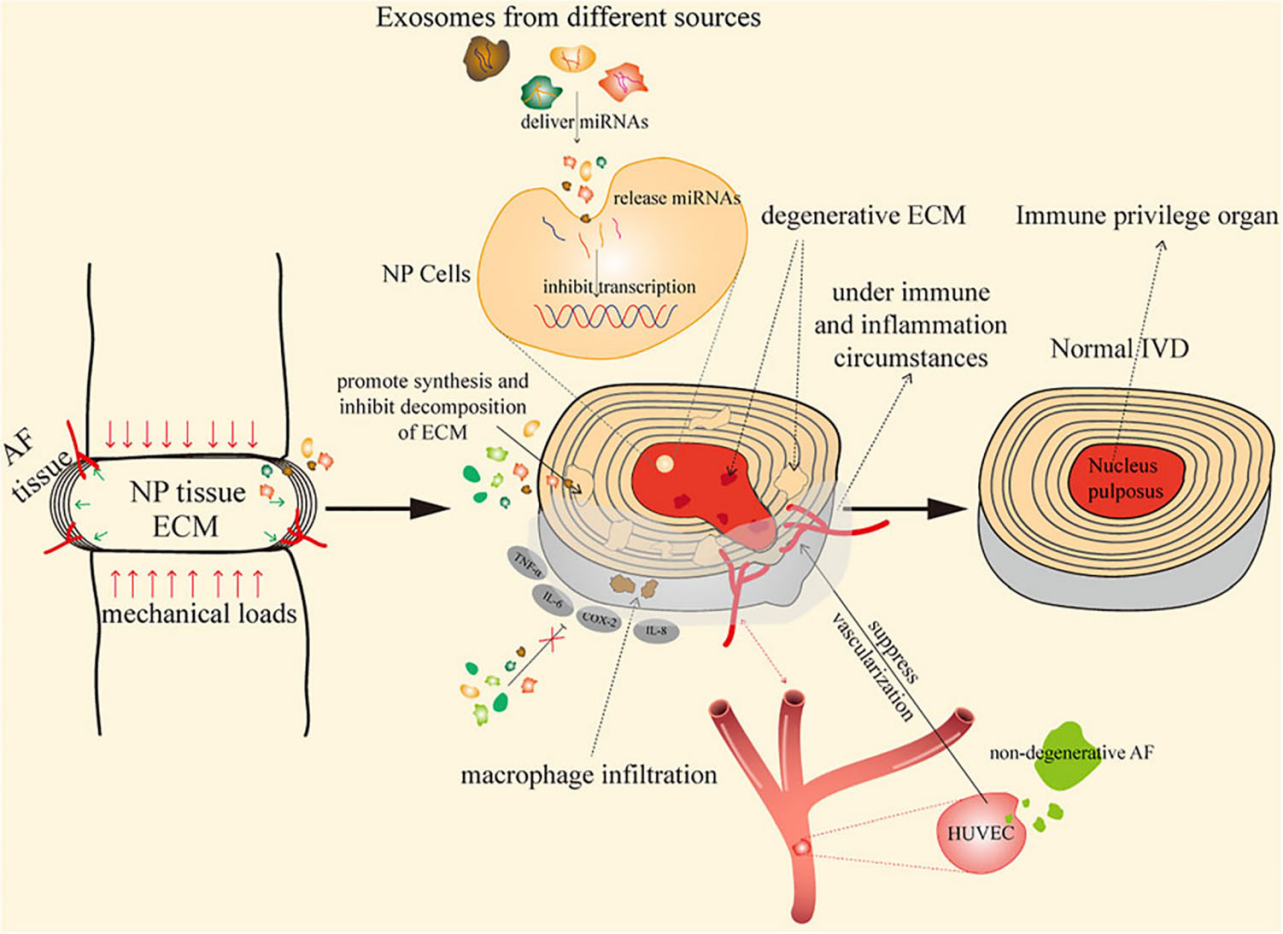
Schematic representation of the role of exosomes from various sources in IVD homeostasis and degeneration. Exosomes deliver miRNAs to NP cells, ECM synthesis and inhibiting its degradation. Under mechanical load and inflammatory conditions, exosomes modulate immune responses, suppress vascularization, and reduce macrophage infiltration, thereby preserving the immune privilege of the disc. These processes help maintain normal IVD structure and function, highlighting the therapeutic potential of exosome-based approaches for preventing or reversing disc degeneration. Adapted from Li et al., 2022, Exosomes Immunity Strategy: A Novel Approach for Ameliorating Intervertebral Disc Degeneration, Frontiers in Cell and Developmental Biology, CC BY 4.0. https://doi.org/10.3389/fcell.2021.822149

Exosomes are involved in rescuing IVDD and are crucial in preserving disc homeostasis [64]. They contain bioactive molecules such as messenger RNA (mRNA), long non-codingRNA (lncRNA), circRNA, DNA, lipids, and proteins, which can be transferred to target cells to regulate gene expression and cellular functions [65]. In the context of disc homeostasis, exosomes derived from stem cells or other sources can restore mitochondrial homeostasis, inhibit disc degeneration, and exert antioxidative effects within the IVD. By delivering essential molecules and signalling factors, exosomes contribute to maintaining the balance of cellular activities, ECM synthesis, and cell survival, ultimately supporting the health and function of the IVD and promoting its homeostasis [66]. Figure 4 depicts the mechanisms of exosomes in the treatment of IVDD.

### Boosting autophagy

Autophagy initiates the breakdown of dysfunctional cellular components, relying on a coordinated network for waste management. Fragmented materials are transported via the lysosomal/exosomal secretory pathway, where lysosomes degrade waste and exosomes expel specific products, maintaining cellular homeostasis by efficiently eliminating debris. Exosomes enhance autophagy in the disc by improving the intracellular vesicular system and regulating the environment, stimulating the Akt-mTOR pathway to maintain homeostasis in NPCs [67, 68]. Additionally, exosomes derived from cartilage end plate MSCs can also regulate autophagy and ECM metabolism in NPCs by delivering their contents [69]. By modulating the Akt-mTOR signaling pathway, MSC-derived exosomes not only reduce inflammation but also restore extracellular matrix homeostasis, thereby offering a promising therapeutic strategy for IVDD management [70].

### Inhibiting inflammatory response pathways

Exosomes play a crucial role in regulating inflammation associated with IVDD by modulating proinflammatory chemokines and cytokines in disc cells. They act as transporters of soluble mediators, influencing TLR cascades and NF-κB pathways, thus affecting immune responses. Exosomes contain various RNA species, including mRNAs and noncoding regulatory RNAs, which can regulate inflammatory pathways. Their release may reduce immune responses, contributing to disc homeostasis and preventing degeneration. [37, 71]. Evidence also suggests that exosomes derived from MSCs can combine miRNA and corresponding messenger RNA functions to inhibit the activation of inflammatory factors and the inhibition of cell autophagy, thereby reducing the series of inflammatory reactions that could help in delaying IVDD [65].

### Promoting extracellular matrix synthesis

Exosomes act as messengers in the IVD, promoting the production of type II collagen and aggrecan, essential for maintaining healthy disc structure. They deliver bioactive cargo like miRNAs, proteins, and transcription factors, influencing gene expression and cellular processes in IVD cells [57]. Notably, stem cell-derived exosomes enhance IVD cell proliferation, inhibit apoptosis, and promote ECM synthesis, restoring balance in the disc. They regulate matrix-building factors like TGF, maintaining the crucial balance between ECM creation and breakdown, which is vital for disc health and function [72].

Additionally, Exosomes are known to prevent apoptosis, stimulate anti-inflammatory responses, and inhibit matrix degradation, supporting ECM preservation in the IVD. They also influence gene transcription, impacting cell division and survival, essential for ECM production and maintenance [73, 74]. A recent investigation suggests that MSC exosomes can modulate the differentiation and function of chondrocytes, resulting in enhanced synthesis of the cartilage matrix, a pivotal component for maintaining structural integrity in OA. Given the shared presence and function of chondrocytes in both OA and IVDD, these findings raise the intriguing possibility that MSC-derived exosomes might offer a restorative strategy for IVDD as well [75]. Furthermore, exosomes from dermal fibroblasts enhance protein production for ECM organization and remodelling, indicating their broader role in regulating the ECM microenvironment [76].

## Exosome therapy and translational research

In a significant advancement for IVDD therapy, a recent study explored the potential of cartilage end plate stem cells (CESCs) for disc repair. It showed that CESCs can transdifferentiate into NPCs, vital for IVD health, through autocrine exosomes. These exosomes promote CESC migration and transformation into functional NPCs, highlighting the potential of using a patient's own stem cells and exosomes for regenerating damaged disc tissue [77, 78]. A 2017 study found that exosomes from BMSCs and NPCs offer therapeutic benefits for IVDD. BMSC-derived exosomes stimulated NPC proliferation and extracellular matrix production, while NPC-derived exosomes enhanced BMSC migration and differentiation into disc-like cells, indicating a promising cell-free intervention for IVDD [79]. Another study found that exosomes derived from NP cells can induce an NP-like phenotype in rat MSCs, increasing collagen II, aggrecan, and SOX9 levels through the Notch1 pathway. This method is more effective than co-culture techniques, suggesting that NP exosomes are a promising strategy for disc repair and regeneration [80]. Additionally, adipose tissue-derived MSC exosomes exhibit anti-apoptotic properties in neural stem cells, indicating potential for IVDD by inhibiting disc cell death. Further research is required to investigate their specific effects on disc cells [66]. Another in vivo investigation employing a SCI rat model demonstrated enhanced motor function following intravenous administration of human umbilical cord-MSC exosomes. This functional improvement is likely attributed to the exosomes' capacity to promote neuronal survival, as evidenced by elevated BCL2 and reduced Bax and cleaved caspase-9 levels, and to diminish inflammation, reflected by decreased GFAP and IBA1 expression. These observations advocate for human umbilical cord-MSC exosomes as a promising treatment approach for SCI [81].

Chen et al. used an in vitro model simulating the acidic microenvironment of IVDD to study BMSC-exosome on human NPCs. BMSC-exosome enhanced NPCs viability under acidic stress and reduced apoptosis markers, indicating a protective effect against cell death. These findings support further investigation of BMSC-exosome for promoting NPC survival in IVDD's acidic environment [82]. A study by Zhao et al. showed that exosomes released by degenerated NPC cells worsen IVDD in rats by promoting M1 macrophage polarization. These exosomes deliver microRNA-27a-3p (miRNA-27a-3p), which suppresses the PPARγ/NFκB/PI3K/AKT pathway in macrophages, leading to increased pro-inflammatory cytokines (i.e., IL-1β and TNFA) and increased disc degradation. Although exosomes in this case do not provide a therapeutic perspective however the study identifies the potentially harmful role of exosomes in IVDD and suggests the use of targeting exosome-mediated signaling as a therapeutic strategy to reduce inflammation-driven degeneration [83]. Another study says exosomes derived from human urine-derived stem cells have been shown to ameliorate IL-1β-induced IVDD by promoting ECM synthesis in NPCs, as indicated by increased levels of SOX9, collagen II, and aggrecan. These exosomes also reduce apoptosis and endoplasmic reticulum (ER) stress in NPCs by activating the AKT and ERK signaling pathways, thereby supporting cell survival under inflammatory conditions. Furthermore, human urine-derived stem cell exosomes deliver MATN3 protein, which stimulates the TGF-β pathway, enhancing NPC proliferation and ECM production, and highlighting their therapeutic potential for IVDD [84].

## Role of exosomes as a delivery vehicle

**Fig. 5:**
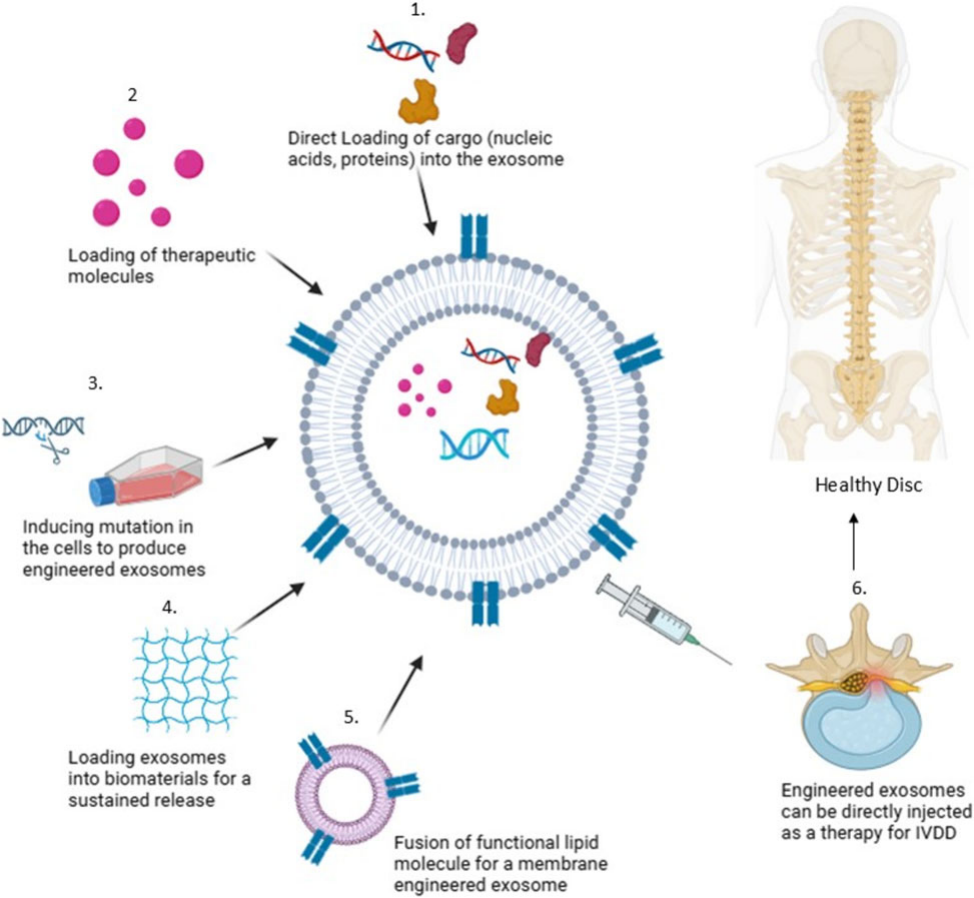
Exosome engineering strategies for IVDD therapy. The depicted strategies include: (1) direct loading of exosomes with therapeutic cargo, such as nucleic acids or proteins, enabling targeted delivery of specific molecules; (2) inducing mutations in cells to produce engineered exosomes with enhanced therapeutic properties; (3) loading exosomes into biomaterials for sustained release and localized delivery; (4) fusion of functional lipid molecules to engineer the exosome membrane for improved targeting and drug delivery; and (5) direct injection of engineered exosomes for minimally invasive IVDD therapy. (made from Biorender)

Exosomes in their natural form hold immense promise as a cell-free therapeutic approach for various diseases, including multiple sclerosis (MS), IVDD, OA, wound healing, etc. These tiny messengers boast several advantages: excellent biocompatibility, minimal toxicity, and low immune system activation. However, they can be employed independently or combined with other therapies for enhanced efficacy. Exosomes are capable of delivering some miRNAs to NPCs, annulus fibrosus, and cartilage endplate cells, thereby modulating essential cellular processes linked to IVDD. These processes include inhibition of apoptosis of NPCs, promotion of cell proliferation and ECM synthesis, inhibition of inflammatory responses by downregulating pro-inflammatory cytokines and inflammasome activation, and restoration of mitochondrial homeostasis and reduction of oxidative stress in NPCs [46]. For instance, MSC-derived exosomal miR-21 suppresses PTEN and regulates the PI3K/Akt pathway to suppress NPC apoptosis with a high potential to inhibit IVDD progression [85]. Other miRNAs, such as miR-532-5p, can suppress TNFA-induced apoptosis, ECM degradation, and fibrosis in NPCs [86]. Furthermore, placental MSC exosomes carrying miR-4450 was reported to alleviate apoptosis and inflammation by regulating ZNF121 expression [87]. Besides, NPC-derived exosomal miR-27A can suppress ECM degradation by targeting MMP-13, and circular RNA (circRNA_0000253/miRNA141-5p signaling, which has been implicated in resisting matrix degradation and cell apoptosis in degenerative discs [88, 89]. All of these findings refer to the complicated therapeutic potential of exosome-mediated miRNA delivery in the modulation of IVDD pathological processes. Modifying an exosome may involve encapsulating it within biomaterials. This not only protects them but also enables sustained release, overcoming limitations associated with their short lifespan and instability. Moreover, scientists are pioneering a strategy that utilizes a sophisticated injectable delivery system. A research explores a hydrogel, derived from hyaluronic acid, which acts as a scaffold and carries a powerful cargo: exosomes manufactured by bone marrow MSCs. They appear to orchestrate a multi-pronged attack on the degenerative disc. By promoting the health of disc cells, stimulating the production of a robust ECM, and quelling inflammation and pyroptosis (a form of cell death), this innovative approach paves the way for a groundbreaking avenue in disc regeneration [57]. Preclinical studies suggest this approach is effective, with engineered exosomes demonstrating a favorable safety profile and surpassing synthetic nanoparticles due to their natural origin, minimal immune response, and ability to cross biological barriers. Figure 5 below [90] depicts the different ways to engineer exosomes, hence increasing the efficiency of cargo delivery.

## Challenges in exosome therapy

Exosome therapy for IVDD faces several challenges that must be addressed for effective clinical application. Currently, the therapy is in its early stages, with limited clinical data on its efficacy and safety, complicating the transition to human trials [91]. The complex processes of isolating and purifying exosomes can lead to variability in quality and quantity, potentially resulting in inconsistent therapeutic outcomes [92]. Additionally, delivering exosomes to damaged discs is challenging due to their small size and the body's clearance mechanisms [93]. Regulatory hurdles also pose significant delays in clinical translation, as exosome-based treatments undergo stringent scrutiny. Furthermore, producing exosomes at a clinically viable scale can be expensive, limiting accessibility. The lack of standardized protocols for production and administration contributes to inconsistent results across studies [94].

## Engineered exosomes and future prospects

Recent advancements in exosome engineering have opened new avenues for targeted delivery of therapeutics, particularly in the context of IVDD. One of the most important factors in the optimization of exosome therapies is the method by which therapeutic molecules are loaded into exosomes. Passive loading techniques, such as incubation and freeze–thaw cycles, rely on natural diffusion or membrane permeability to deliver bioactive molecules like miRNAs or small molecules into exosomes. While simple and comparatively non-disruptive, these methods have lower loading efficiency. In contrast, active loading techniques, including sonication, electroporation, and extrusion, enhance membrane permeability to achieve higher cargo encapsulation efficiencies. For instance, electroporation was used to load siRNAs into exosomes for gene silencing applications, while extrusion has demonstrated higher efficiency for the encapsulation of small molecules like paclitaxel into MSC-derived exosomes [95]. Chamberlain et al. in 2023 investigated the impact of a mesenchymal stromal cell's microenvironment on the RNA content of its exosomes. It was revealed that altering the microenvironment significantly impacts the therapeutic cargo of exosomes, suggesting a strategy for engineering exosomes with enhanced properties for various diseases. In IVDD therapy, this approach could involve enriching exosomes with specific RNAs that promote disc repair by optimizing the MSC culture environment, highlighting a promising direction for targeted exosome therapies [96]. These engineering approaches are particularly relevant to the treatment of IVDD, in which therapeutic molecules like miRNAs or CRISPR-Cas9 elements must be precisely delivered. Active loading methodologies can be used to deliver miRNAs against inflammatory mediators or ECM breakdown into MSC-derived exosomes, and this will enhance their therapeutic value for disc curing. Passive loading methodologies can also be used for hydrophilic drugs, which can freely diffuse into exosomes under culture [95]. Future research should focus on identifying optimal MSC microenvironments and translating these findings into preclinical IVDD models [96]. While the field of engineered exosomes holds immense promise for targeted therapies, the provided study by Dubey et al. doesn't directly address IVDD treatment [97]. Their research explores a powerful approach for engineering exosomes as delivery vehicles for CRISPR-Cas9 gene-editing machinery. This development paves the way for various therapeutic applications that could benefit from precise genome editing. The propensity of engineered exosomes for IVDD therapy lies in their ability to deliver therapeutic molecules specifically to disc cells. A bone regeneration study using urine stem cell exosomes in a hydrogel suggests a similar approach for IVDD. Both therapies rely on tissue regeneration and exosomes' ability to repair tissues. The bone study's exosome-hydrogel combo promoted bone growth and blood vessel formation. Adapting this with IVDD-specific exosomes could stimulate disc cell and matrix regeneration, offering a promising treatment strategy [98]. A study exploring collagen/chitosan scaffolds loaded with neural stem cell exosomes for brain injury holds promise for IVDD treatment. In the context of IVDD, exosome-loaded scaffolds could be designed using NPCs or other relevant sources. These scaffolds could deliver exosomes in a controlled manner, promoting the regeneration of NPCs and enhancing the disc's ECM. This approach offers a promising therapeutic strategy for IVDD [80, 99]. Table 2 below compares exosome therapy with cell therapy, gene therapy, and medications [99, 100]

**Table 2:** Comparison of Exosome therapy with conventional therapies

Feature	Exosome therapy	Cell therapy	Gene therapy	Medications
Mechanism of action	Delivers bioactive molecules for cell growth, repair, and inflammation modulation	Introduces stem cells for direct tissue regeneration	Modifies the genetic makeup of cells for therapeutic protein production	Primarily targets symptoms like pain and inflammation
Minimally invasive	Often involves injection into the disc space	Requires cell isolation, expansion, and injection	May involve viral vectors or complex delivery methods	Typically oral or topical administration
Immunogenicity	Generally low due to minimal cellular material	Potential for immune response to foreign cells	Potential for immune response to viral vectors or modified genes	Varies depending on the medication
Scalability	Easier to isolate and scale up exosome production compared to cell expansion	Scaling up cell production can be time-consuming and expensive	Gene therapy technology is still under development for large-scale production	Mass production is generally feasible
Targeted effects	Can deliver specific molecules to target various cellular processes	Cell differentiation and function within the disc environment can be unpredictable	Gene targeting can be precise but may have unintended consequences	Primarily targets specific symptoms
Long-term effects	Potential for sustained therapeutic effects through delivered molecules	Cell survival and functionality within the disc may diminish over time	Gene therapy could offer permanent correction, but long-term safety needs monitoring	Effectiveness may diminish over time, requiring repeated administration

## Summary

This review explores the potential of exosome therapy as a novel treatment for IVDD. IVDs are essential for spinal support and mobility but are prone to degeneration due to age, strain, and genetic factors, leading to pain and disability. Traditional treatments primarily focus on pain management, while exosome therapy offers a regenerative approach by utilizing small vesicles that transport proteins and RNA to stimulate tissue repair and reduce inflammation. It is highlighted that exosomes derived from MSCs can promote the regeneration of NPCs and maintain homeostasis within the disc environment. Despite the promising therapeutic potential, there are challenges such as optimizing isolation methods, ensuring safety, and scaling production for clinical use. There is an urgent need for further research to address these hurdles and explore the engineering of exosomes for targeted delivery. Overall, this innovative therapy could lead to effective, minimally invasive treatments for IVDD, enhancing patient outcomes and quality of life.

## Data Availability

The data that supports the findings in this study are available from the corresponding authors upon reasonable request.
